# Screening high-risk Veterans for cirrhosis: taking a stepwise population health approach

**DOI:** 10.1186/s12913-025-12216-8

**Published:** 2025-01-29

**Authors:** Jonathan Dounel, Carolyn Lamorte, Heather Patton, Ponni Perumalswami, Heather McCurdy, Nicole J. Kim, Lauren A. Beste, Dawn Scott, Jessimarie Casey, Patrick Spoutz, Linda Chia, Yiwen Yao, Elliott Lowy, Sandra Gibson, Timothy R. Morgan, Shari S. Rogal

**Affiliations:** 1 VA San Diego Healthcare System, San Diego, CA USA; 2 University of California San Diego, La Jolla, CA USA; 3 VA Center for Health Equity Research and Promotion, Pittsburgh, PA USA; 4 VA Ann Arbor Healthcare System, Ann Arbor, MI USA; 5 VA Center for Clinical Management Research (CCMR), VA Ann Arbor Healthcare System, Ann Arbor, MI USA; 6 Division of Gastroenterology, University of Washington, Seattle, WA USA; 7 VA Puget Sound Health Care System Seattle Division, Seattle, WA USA; 8 Division of General Internal Medicine, Department of Medicine, University of Washington, Seattle, WA USA; 9 VA Central Texas Healthcare System, Temple, TX USA; 10 VA Pittsburgh Healthcare System, Pittsburgh, PA USA; 11 Pharmacy Benefits Management, VA Northwest Health Network (VISN 20), Vancouver, WA USA; 12 VA Salt Lake City Health Care System, Salt Lake City, UT USA; 13 VA Long Beach Healthcare System, Long Beach, CA USA; 14 Department of Medicine, University of California, Irvine, CA USA; 15 Departments of Medicine and Surgery, University of Pittsburgh, Pittsburgh, PA USA

**Keywords:** Veterans, Screening, Cirrhosis, Primary care, Elastography

## Abstract

**Background:**

Because cirrhosis is often unrecognized, we aimed to develop a stepwise screening algorithm for cirrhosis in the Veterans Health Administration (VHA) and assess this approach’s feasibility and acceptability.

**Methods:**

VHA hepatology clinicians (“champions”) were invited to participate in a pilot program from June 2020 to October 2022. The VHA Corporate Data Warehouse was queried to identify Veterans with possible undiagnosed cirrhosis using Fibrosis-4 (FIB-4) ≥ 3.25 and at least one risk factor for liver disease (e.g., obesity), and generate an age-stratified sample. Champions at four sites reviewed charts to confirm eligibility and contacted Veterans to offer further evaluation with elastography. Feasibility was defined as protocol implementation with completion of at least one elastography test and acceptability was defined based on Veteran- and clinician-reported surveys. Participation in the program, patient outcomes, adaptations to the protocol, and implementation barriers were also assessed.

**Results:**

Four sites were able to implement the screening protocol. Adaptations included type of outreach (primary care vs. hepatology, phone vs. mail) and type of elastography used. One site chose to refer patients with clear evidence of cirrhosis directly to hepatology (*n* = 12) rather than to elastography. Key implementation barriers included staffing, primary care provider (PCP) comfort with interpreting and communicating results, and appointment availability during the COVID-19 pandemic. Of 488 patients whose charts were reviewed, 230 were excluded from outreach based on predefined criteria (e.g., advanced cancer, prior or current referral to hepatology). Champions and PCPs attempted to contact 165 of 246 Veterans who were deemed eligible for evaluation with elastography. Among 53 Veterans who completed elastography, 22 (42%) had findings consistent with significant fibrosis and were referred to hepatology. Clinicians and Veterans reported high acceptability of the program on surveys (80% of Veterans who completed survey).

**Conclusions:**

This pilot demonstrated the feasibility, acceptability, and challenges of a multisite approach to cirrhosis screening.

**Supplementary Information:**

The online version contains supplementary material available at 10.1186/s12913-025-12216-8.

## Background

Chronic liver disease, including cirrhosis, is under-diagnosed in the general population [1]. Patients and providers often remain unaware of cirrhosis until the onset of costly and life-threatening cirrhotic decompensation events or hepatocellular carcinoma (HCC). Early identification of cirrhosis offers the promise of preventing complications and reducing mortality, but it is unclear how to best implement such programs [2]. In practice, providers often rely on abnormal liver blood tests that lack sensitivity and specificity for cirrhosis [3, 4]. More accurate screening approaches have included different combinations of risk calculators, lab testing, and imaging [3, 5]. Among highly accessible and low-cost approaches is the Fibrosis-4 (FIB-4) score, which includes age, alanine transaminase (ALT), aspartate aminotransferase (AST), and platelet count [6]. FIB-4 > 3.25 has a high positive predictive value for cirrhosis [7], making it a promising tool for population-based screening [8–10]. More recently, investigators have added vibration controlled transient elastography (VCTE) [11, 12] as a second step that has been recommended by guidelines [13, 14] and applied in stepwise programs that use FIB-4 to identify patients with cirrhosis [3, 4, 15]. However, such approaches have not been broadly implemented in the United States (US).

The Veterans Health Administration (VHA) is the largest provider of cirrhosis care in the US. Nearly half of patients who receive care at VHA medical centers (“sites”) have risk factors for liver disease, yet estimates suggest that only 10% of Veterans with risk factors and high FIB-4 are diagnosed with cirrhosis [16, 17]. VHA does not currently employ standard population-level, risk-based screening for cirrhosis. Given the estimated prevalence of undiagnosed cirrhosis, and the growing need to triage patients with metabolic-associated steatotic liver disease (MASLD), the aims of this pilot project were to: 1) develop a stepwise screening algorithm for cirrhosis in VHA and 2) assess this approach’s feasibility and acceptability.

## Methods

### Site identification

This pilot was initiated by VHA’s hepatology leadership team in the National Gastroenterology and Hepatology Program (NGHP). Hepatology clinicians (“champions”) from 11 sites initially expressed interest in participating. Champions met biweekly to develop a written project protocol that included a screening algorithm, exclusion criteria for chart reviews, proposed pathway for patient outreach and flow from primary to specialty care, template for introducing the project to primary care (PC) leadership, brief education for primary care physicians (PCPs), and resources for healthy liver education to participating patients. Due to limited availability during the COVID-19 pandemic, only four of 11 sites attempted to implement the protocol; all four were able to enroll patients. NGHP and participating sites’ institutional review boards deemed this project to be quality improvement (QI).

### Screening algorithm and patient identification

The project team selected a screening algorithm by consensus, after careful review of the literature (Additional File 3) and discussion with colleagues who had implemented similar approaches in other healthcare systems. FIB-4 was selected because of its widespread use in VHA, the fact that it has been extensively validated [6, 18, 19], employs standard labs that are available for nearly all Veterans in care, and is a standard part of the workflow of VHA providers.

The VHA Corporate Data Warehouse was queried to identify potentially eligible patients from each participating site. Eligibility criteria included: 1) age 45–75 years; 2) FIB-4 ≥ 3.25 (capped at age 65 years) [7]; and 3) at least one risk factor for liver disease. These factors included either obesity (body mass index ≥ 30 kg/m^2^) or the presence of two outpatient or one inpatient validated International Statistical Classification of Diseases and Related Health Problems (ICD)−10 codes for diabetes mellitus (DM), alcohol use disorder (AUD), or hepatitis C infection (HCV) [20–27]. Veterans were excluded if they had 1) prior diagnosis codes for cirrhosis or its complications; 2) an outpatient hepatology visit in the last year; 3) codes for hospice; or 4) prior liver transplantation (based on Current Procedural Terminology (CPT) codes).

### Chart review

The coordinating team generated a sample of Veterans, stratified by age decile. Champions reviewed the charts of Veterans receiving care in their facilities to determine appropriateness for further testing. Veterans were excluded from further evaluation based on criteria agreed upon by project champions: 1) limited life expectancy due to comorbidities (e.g., advanced congestive heart failure or cancer); 2) alternative explanations for a high FIB-4 score (e.g., non-hepatic causes of thrombocytopenia), 3) contraindications to elastography relevant during the project years that were subsequently removed in the United States in 2023 (e.g., pregnancy, automated implantable cardioverter defibrillator placement, ascites or anasarca); 4) evidence of active liver care in the record, or 5) no longer receiving VHA care. Champions tracked reasons for exclusion in a structured database.

### Local education and engagement

An informational slide deck with an overview of cirrhosis in VHA, rationale for screening and a pathway to diagnosis and linkage to specialty care was created by champions and used at all four sites. Using a pragmatic approach, champions presented the proposed pathway to local (PC) leadership and then collaboratively developed a process for identifying and approaching eligible patients, adapted to local context. The coordinating team tracked each site’s progress and barriers from June 2020 to October 2022 using emails and notes collected during project meetings.

### Veteran engagement and follow-up

Veterans were contacted by phone or mail to offer elastography, including FibroScan® in three sites and ultrasound elastography in one. VCTE exams were performed in alignment with best practices (trained examiner, requisite fasting 3 h prior to examination, Interquartile range/Median (IQR/M)% < 30 and interpretation by an experienced specialist provider). The IQR/M ratio was used as a necessary indicator of VCTE quality [28], where the ratio indicates the consistency of measurement, defined as IQR of kPa measures divided by the median of these measures (M), with a percentage of < 30% applied as the threshold for consistency and quality [19, 29].

Veterans presenting for elastography followed recommended testing protocols (e.g., fasted for at least three hours prior) and were provided with a VHA Healthy Liver Education document and relevant VHA heath education materials about MASLD, viral hepatitis, alcohol-related liver disease, or cirrhosis at the appointment. Results were placed in the medical records and reviewed with the patients. The study team collected median FIB-4, liver stiffness measurements (kPa or m/s), IQR%, and CAP scores when applicable and available. Veterans with significant fibrosis, defined by consensus as median liver stiffness measurements ≥ 8 kPa (kilopascals) or F2 or higher fibrosis on ultrasound elastography, were offered specialty care consultation with gastroenterology or hepatology.

### Outcomes

The study team tracked implementation barriers, adaptations to the protocol, and patient completion and results over time. Feasibility was defined by the ability to institute the protocol in a hospital and screen at least one patient through this pathway. Acceptability was defined as at least 65% of patients verbally agreeing to elastography and an average satisfaction score of at least 3 on a 5-point Likert scale as reported by patients and providers. Participating Veterans were mailed surveys to assess their satisfaction with steps of the elastography process (from outreach to results communication). Survey items were rated on a 5-point Likert scale from 1-Very Dissatisfied/Strongly Disagree to 5-Very Satisfied/Strongly Agree. Other questions inquired about health literacy and the impact of receiving results (Supplementary File 1). Provider surveys asked about acceptability of and challenges with implementing the screening protocol (Supplementary File 2).

### Analysis

Demographic characteristics, risks for liver disease, and test results for Veterans who completed staging elastography were aggregated by facility and overall. Summary statistics were used to describe clinical and demographic characteristics and responses to the surveys, using means and medians for continuous variables, and frequencies and percentages for categorical variables. Chi-square testing was used to compare elastography completion rates for patients who received outreach from a PCP versus a champion.

## Results

### Facility participation

Between June 2020 to October 2022 champions from four sites (Ann Arbor, Pittsburgh, Puget Sound, and San Diego), including hepatologists, nurse practitioners, nurses, and trainees, completed 488 chart reviews. Barriers for the other seven sites that had initially expressed interest in participating included staffing changes and turnover, scheduling during the COVID-19 pandemic, and lack of local site leadership support. The four participating sites all had hepatology providers onsite.

### Chart review results

**Fig. 1:**
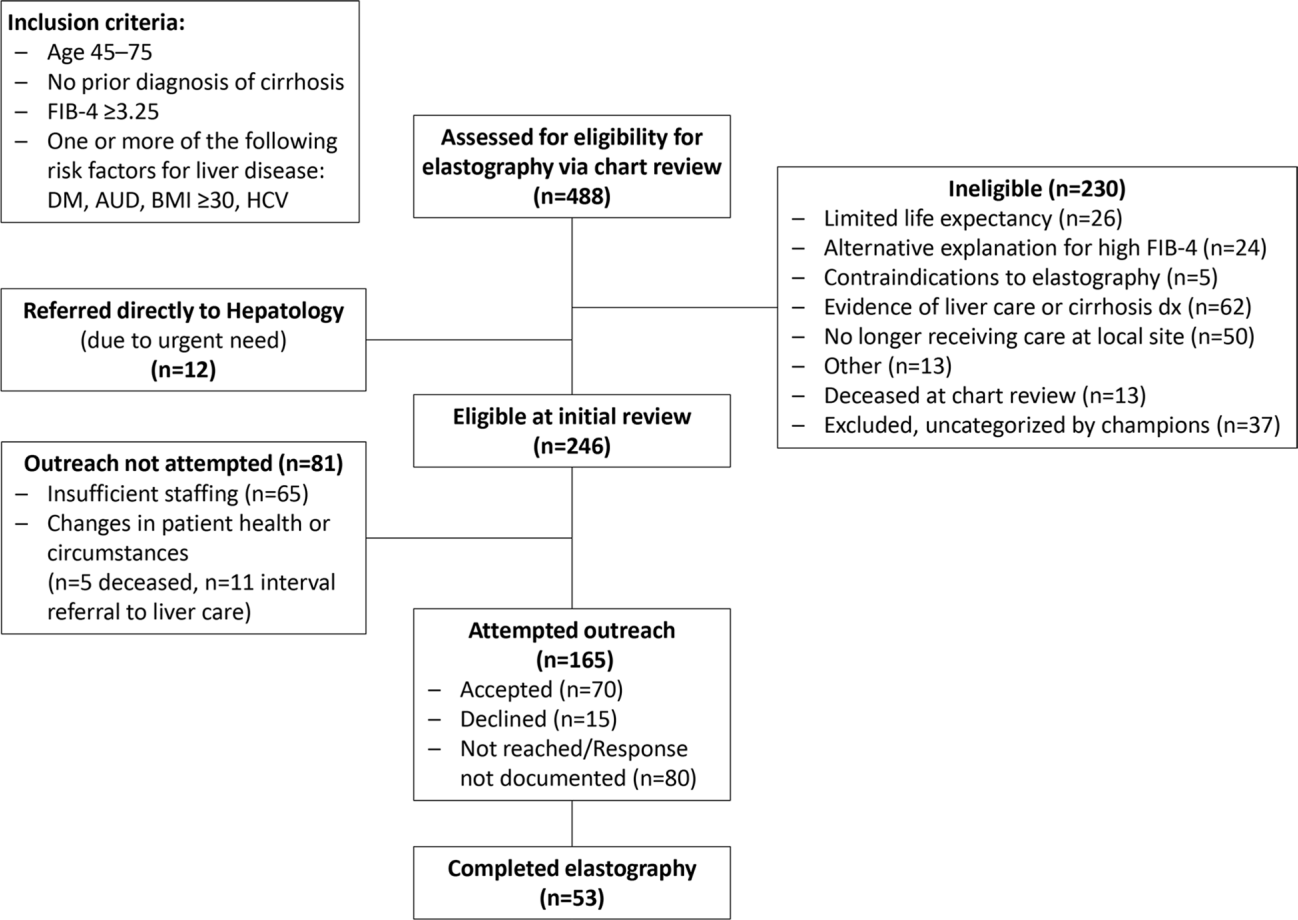
Patient flow diagram for elastography screening

Figure 1 illustrates the flow of patients through the study. Of 488 Veterans whose charts were reviewed, 465 (95%) were male, 298 (61%) were non-Hispanic white, 105 (22%) were non-Hispanic Black or African American, 31 (6%) had Unknown race/ethnicity, 29 (6%) were Hispanic or Latino, 10 (2%) were Asian, seven (1%) identified as more than one race, six (1%) were Native Hawaiian or Other Pacific Islander, and two (0.4%) were American Indian or Alaska Native. Of these 488 Veterans, the average Charlson Comorbidity Index (CCI) was 2.06.

Slightly more than half of Veterans (*n* = 246) were considered eligible after chart review, with 230 deemed ineligible based on project exclusions: limited life expectancy (*n* = 26), alternative explanations for a high FIB-4 score (*n* = 24), contraindications to elastography (*n* = 5), in liver care/diagnosis of cirrhosis (*n* = 62), no longer receiving care at local site (*n* = 50), death between data collection and chart review (*n* = 13), other (*n* = 13) or uncategorized (*n* = 37) reasons.

### Adaptations and barriers

**Fig. 2:**
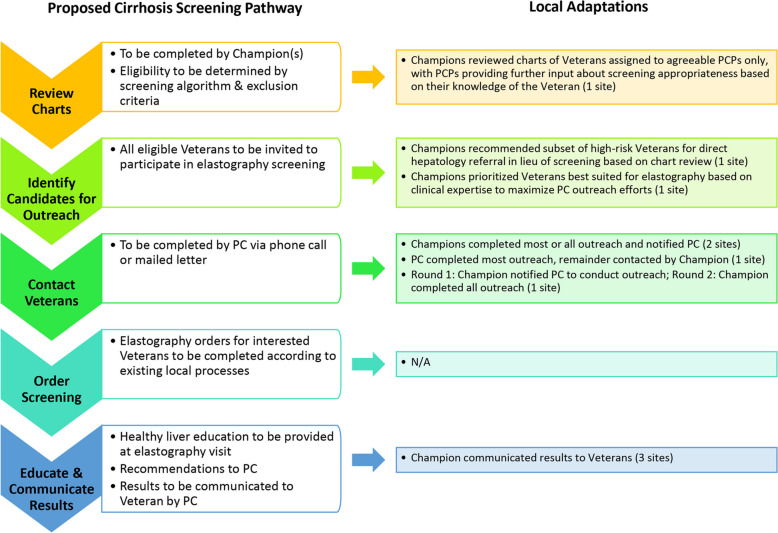
Local implementation of screening pathway

Sites adapted the process to fit local context, including adjustments to who was doing outreach, type of outreach, and one site opting to recommend the direct referral of 12 patients to liver care based on having high risk of cirrhosis and clear need for urgent hepatology management (Fig. 2).

### Patient outreach

Out of 246 eligible candidates, we attempted to invite 165 (67%) Veterans for elastography; 85 (52%) were reached by the team and had documented responses. Among those with documented receipt of outreach, 70 of 85 agreed to testing (82%) and 15 declined testing, meeting our goal of at least 65% patient program acceptance, among those who received documented outreach. Subsequently, 53 of these 70 underwent testing (76%). We did not attempt outreach with 81 patients due to insufficient staffing or changes in patient health or circumstances.

The preferred method for patient outreach varied by site and was not significantly associated with participation. Primary care physician (PCP) outreach resulted in a 47% (9/19) elastography completion rate compared to a 30% (44/147) completion rate when outreach was conducted by champions (*P* = 0.124, based on chi-square test). One patient received outreach from both PCP and champion.

### Patients who completed elastography

Among the patients who completed staging elastography (*n* = 53), 49% were 65–74 years old, 92% were men, the average CCI was 1.40, and the median FIB-4 was 4.54, with a range of 3.25–10.03. The most common risk factor in this cohort was obesity (53%), followed by AUD (51%), DM (35%) and HCV (8%) (Table 1).

**Table 1:** Demographic characteristics of 488 Veterans with risk factors for liver disease and high FIB-4 scores

	**Total Charts Reviewed**	**Direct Hep Referral (advised) at Chart Review**	**Not Eligible**	**Eligible**	**Outreach Not Attempted**	**Outreach Attempted**	**Patient Accepted**	**Patient Declined**	**Patient Not Reached/** **Response Not Tracked**	**Completed** **Screening**
**Characteristics**	**(*n* = 488)**	**(*n* = 12)**	**(*n* = 230)**	**(*n* = 246)**	**(*n* = 81)**	**(*n* = 165)**	**(*n* = 70)**	**(*n* = 15)**	**(*n* = 80)**	**(*n* = 53)**
**Age **(n, %)										
45–54	99 (20%)	2 (17%)	49 (21%)	48 (20%)	14 (17%)	34 (21%)	15 (21%)	1 (7%)	18 (22%)	11 (21%)
55–64	164 (34%)	4 (33%)	74 (32%)	86 (35%)	31 (38%)	55 (33%)	21 (30%)	8 (53%)	26 (33%)	16 (30%)
65–74	225 (46%)	6 (50%)	107 (47%)	112 (45%)	36 (45%)	76 (46%)	34 (49%)	6 (40%)	36 (45%)	26 (49%)
**Gender** (n, %)										
Male	465 (95%)	12 (100%)	220 (96%)	233 (95%)	80 (99%)	153 (93%)	65 (93%)	13 (87%)	75 (94%)	49 (92%)
Female	23 (5%)	0 (0%)	10 (4%)	13 (5%)	1 (1%)	12 (7%)	5 (7%)	2 (13%)	5 (6%)	4 (8%)
**Risk Factor** (n, %)										
DM	180 (37%)	6 (50%)	91 (40%)	83 (34%)	26 (32%)	57 (34%)	25 (36%)	4 (27%)	28 (35%)	18 (35%)
Obesity	191 (39%)	7 (58%)	84 (37%)	100 (40%)	35 (43%)	66 (40%)	24 (34%)	7 (47%)	24 (30%)	28 (53%)
AUD	274 (57%)	7 (58%)	123 (53%)	144 (59%)	46 (57%)	100 (61%)	40 (57%)	8 (53%)	51 (64%)	27 (51%)
HCV	56 (11%)	1 (8%)	26 (11%)	29 (12%)	9 (11%)	20 (12%)	8 (11%)	2 (13%)	10 (13%)	4 (8%)
**FIB-4** (median, range)	5.21 (3.25–25.41)	7.12 (5.58–10.0)	5.08 (3.25–25.41)	5.26 (3.25–16.98)	5.75 (3.30–10.65)	4.67 (3.25–16.98)	5.16 (3.25–16.98)	5.13 (3.51–9.83)	4.51 (3.25–14.94)	4.54 (3.25–10.03)
**CCI** (mean ± SD)	2.06±1.81	2.5±1.88	2.4±1.87	1.72±1.70	1.59±1.57	1.78±1.76	1.56±1.58	1.87±1.68	1.95±1.92	1.40±1.62

### Elastography results and hepatology referral

Table 2 includes the clinical characteristics and outcomes of the 53 Veterans who completed elastography tests. Fifty of the elastographies were done by VCTE and three by ultrasound; 22 had kPa < 6 (consistent with F0-F1 disease), 15 had scores of 6–10.5 (consistent with F2 disease), four had scores of 10.5–16 (consistent with F3 disease), and 12 had scores ≥ 16 (consistent with F4 disease). Among those who completed VCTE (*n* = 50), the median stiffness was 6.6 kPa, with a range of 3.7–74.8 kPa. In total, 22 of 53 patients had kPa ≥ 8 (our prespecified cutoff). Of these 22 Veterans, 15 (71%) had been seen by hepatology at the time of follow-up chart review, one had been sent for MRE for further risk-stratification, two had died due to non-liver-related events, and four had not yet attended hepatology follow-up. At the site where champions opted to recommend that primary care directly refer patients to hepatology (based on clear chart indications), the champion communicated with primary care clinicians for 12 high-risk Veterans. At the time of chart reviews, five Veterans were referred to hepatology by primary care; three declined referral, three had no documentation of referral discussion, and one had died.

**Table 2:** Elastography results (*n* = 53)

	**VCTE (*n* = 50)**	**U/S elastography (*n* = 3)**	**Total (*n* = 53)**
**Number of risk factors for liver disease** (count of diabetes, obesity, AUD, and HCV)			
< 2	30 (60%)	2 (67%)	32 (60%)
2	18 (36%)	1 (33%)	19 (36%)
> 2	2 (4%)	0	2 (4%)
**CCI** (mean±SD)	1.42±1.65	1.00±1.00	1.40±1.62
kPa (median, range)	6.6 (3.7–74.8)	n/a	n/a
IQR (median %, range)	14 (3–29)	n/a	n/a
CAP (median, range)	296 (199–400)	n/a	n/a
Liver Stiffness			
kPa < 6 (F1)	21 (42%)	1 (33%)	22 (42%)
kPa 6–10.5 (F2)	13 (26%)	2 (67%)	15 (28%)
kPa 10.5–16 (F3)	4 (8%)	0	4 (7%)
kPa ≥ 16 (F4)	12 (24%)	0	12 (23%)
Referred to hepatology (n, %)	21 (42%)	1 (33%)	22 (44%)
Seen in hepatology (n, %)	15 (30%)	0	15 (28%)

### Patient satisfaction/patient survey results

Among 53 Veterans who completed elastography testing, 17 (32%) responded to a mailed feedback survey, and 15 of those (88%) answered the question about acceptability. Eighty percent (12/15) were “satisfied” or “very satisfied” with their participation in the pilot. Most respondents were satisfied with all steps of the screening process and cited few barriers. However, some respondents reported feeling “neutral” about statements describing appointments as easy to attend (18%), the elastography procedure as comfortable (12%) and test results easily understandable (18%). All patients either agreed or strongly agreed they would recommend the screening to others; 82% (14/17) reported the screening procedure changed how they think about liver health, and 59% (10/17) report planning to take action to improve liver health post-screening. Using free text, Veterans reported that they planned to make the following changes: following a Mediterranean diet, decreasing alcohol intake, and focusing on weight loss.

### Provider survey results

Of 117 PCPs with Veterans contacted through this program, 19 responded to surveys (16%). For the task of interpreting elastography results, 58% (11/19) reported “low confidence” and 47% (9/19) reported “low confidence” with talking to patients about results. The most highly endorsed barriers to program participation were reported as: Provider/staff time (63%); Patient willingness to participate (47%); Provider knowledge about liver disease (32%) and Communication between providers (32%). Only 52% of responding providers were aware of this QI effort. Of these 10 providers who were aware of the QI effort, 78% reported that the program helped them to better identify Veterans with possible cirrhosis, 33% reported improved communication with specialists. Six of nine PCPs supported wider implementation of this initiative, and three were neutral about scaling this approach.

### Barriers to implementation

Based on comprehensive review of patient and provider feedback during meetings and through anonymous surveys, four categories of implementation barriers were identified and are summarized in Table 3.

**Table 3:** Barriers to Implementation

Category	Barrier
Patient Factors	Ineligibility due to improvement of labs during chart review Ineligibility due to previous established specialty care Discomfort due to receipt of invitation from unfamiliar provider Intercurrent competing illnesses
Provider Factors	Allocation of time for chart review and/or patient outreach Discomfort with discussion of elastography exam findings with patients Lack of understanding of indication for hepatology referral Lack of response to request for patient outreach/communication
Healthcare System	Ease of communication between patients and providers regarding test results Lack of support from Primary Care leadership Ease of appointment scheduling process for patients
Environment	Interruption of clinical care services imposed by COVID-19 pandemic

## Discussion

It is critical to identify patients with advanced fibrosis and cirrhosis prior to decompensation to prevent morbidity and mortality [2, 30], however developing a process that is efficient and effective for doing so has been challenging. This multisite quality improvement project employed a stepwise approach using two validated screening tools to identify Veterans with undiagnosed cirrhosis. Using FIB-4 score followed by elastography, we developed a feasible process that could be tailored according to site resources and preferences. While the process was acceptable to patients and providers, we also identified barriers to implementation that can inform future population-level cirrhosis identification efforts.

The present study was designed to test the feasibility and acceptability of implementing a simple, pragmatic approach for evaluating a smaller group of patients who would potentially benefit from hepatology consultation. This was offered as an alternative to the current approach of using FIB-4 alone to identify potential patients for referral. Conversely, this study was not designed to establish the performance of blood-based or elastography-based screening, which has been extensively studied and is described by the recent American Association for the Study of Liver Diseases’ guidance around noninvasive liver disease assessment [19, 31]. Current guidance highlights the need to implement non-invasive testing and the challenges of doing so. One such challenge is that test thresholds may vary across disease etiologies and studies.

Through the collaborative and flexible process that we developed, over half of the patients who were identified through nationally coordinated data were eligible for testing or direct referral to hepatology, and over half who completed elastography met criteria for hepatology referral. Additionally, we identified one patient with HCC, and another had unfortunately died of HCC prior to our chart review. This yield was higher than in other similar population risk-based screening approaches, through which only 12% completed VCTE and 10–13% of these patients were referred to hepatology [15, 32]. Supplementary File 3 summarizes the criteria used by similar studies [3–5, 32, 33], illustrating that our higher FIB-4 threshold may have contributed to the higher yield. Additionally, Veterans in VHA care have increased risk factors for cirrhosis compared to the average US population, related to higher rates of AUD, metabolic syndrome, and HCV [34, 35]. Overall, these findings demonstrate a need to consider staged screening approaches for patients with risk factors for liver disease.

Beyond identifying undiagnosed cirrhosis, risk-based engagement programs offered other benefits, including the opportunity to provide patient and provider education. Veterans were provided with education materials about liver health and specific etiology- or stage-focused materials for MASLD, viral hepatitis, alcohol-related liver disease, or cirrhosis. Most participating Veterans reported that the screening procedure changed how they thought about liver health. Approximately half planned to take action to improve liver health post-screening, suggesting a key benefit of instituting engagement programs. Cirrhosis education interventions have been associated with sustained, increased cirrhosis and disease management knowledge, hepatology engagement, increased HCC surveillance, reduced hepatic complications and hospitalizations [36, 37].

In addition to patient education, this program offered opportunities to educate and engage PCPs about elastography. While the limited response of providers to our survey warrants cautious interpretation, the data suggest that few PCPs were confident in their abilities to refer patients for elastography or discuss subsequent results. Currently, patient referral to hepatology is opportunistic and often not based on need. As cirrhosis prevalence increases with the influx of patients with MASLD and the aging cohort with chronic HCV infection [38, 39], reliable non-invasive detection methods are critical to appropriate triage and management. Identifying an acceptable, cost-effective screening algorithm can enable efficient triage and proactive care that can improve public health.

This pilot project allowed us to identify important lessons prior to scaling an approach to identifying undiagnosed cirrhosis in a high-risk population. While VHA is a unique national healthcare system in the US, other healthcare systems could similarly implement such programs. Key elements of such programs include methods to identify at risk patients, a system for communication and outreach, and linkage to elastography. Electronic medical record systems are nearly universal and simple, inexpensive screening tests like FIB-4 can be calculated using readily available lab tests. Several of the lessons that were learned could be applied outside of VHA. We realized that a more readily updated data source was needed to identify patients. Because we used a one-time data-pull at the start of a project, some information was outdated by the time of outreach, including that some patients had already died or been referred to hepatology prior to review. This was particularly challenging because the work was conducted during the COVID-19 pandemic. To address the time lag between data extraction and review, we have now identified a dashboard that updates daily for these purposes. As is recommended in implementation projects, tailoring to the facility culture and resources helped with implementation. We therefore developed a flexible approach that could be negotiated between site champions and primary care leadership. The feasibility of completing such a project across diverse facilities allowed us to increase the external validity of our findings, though there is a need to tailor this process for sites without specialty hepatology care as a next step.

The role of primary care was an important theme throughout this pilot. Although the method of outreach was not significantly associated with engagement, a greater proportion of patients accepted offers for elastography when contacted by primary care providers who they knew, rather than when they received a call or letter on behalf of the hepatology clinic. We hypothesize that this was due to the rapport established with primary care, but future research is needed to optimize outreach strategies for this population. Several studies in literature have shown that the relationship between the patient and the physician affects shared decision-making, patient adherence, and positively affects treatment outcomes [40–42]. Collectively, these findings suggest that PCPs are important partners in developing effective population-level screening approaches for cirrhosis. While PCPs would be ideal providers to engage patients, they are burdened by addressing multiple health issues within the confines of limited appointment times. Opportunities to provide education and training in interpreting and communicating about elastography could increase the comfort of PCPs, and more work should focus on how to engage nurses and other clinicians in these outreach efforts.

To address implementation barriers identified in this pilot study, we have considered several complementary approaches. Concerted education efforts are needed to improve the identification of cirrhosis in asymptomatic patients. In addition to education, there are several medical record capabilities that could be helpful, including a patient identification system and automated calculation of FIB-4 or other scores that can be flagged with interpretations for providers. Access to non-invasive tests for liver disease staging such as VCTE has increased over time, as has access to tele-hepatology services. At a more societal level, de-stigmatizing diagnoses such as AUD, obesity, and cirrhosis, along with information campaigns to empower patients could help increase engagement. Next steps in the VHA include modifying the protocol for scalability and implementing the approach in other centers using a dashboard.

Despite the many strengths of this approach, there were several notable limitations and caveats. First, this study was not an efficacy study and was not powered to evaluate the diagnostic accuracy of the algorithm; rather this was a proof-of-concept study, demonstrating the feasibility and acceptability, as well as challenges, of offering population-level cirrhosis risk stratification across facilities in VHA. Furthermore, our sample size was smaller after chart review due to a portion of these patients having a change in their status such as improvement in their FIB-4 score or development of other exclusion criteria. Next, Veterans in VHA care may not represent the broader US population. They are more likely to be men and to have other health concerns compared to non-Veteran patients outside of VHA. That said, the comparability of our findings and those in a population-level study in the UK (as described above) illustrates that this approach could be widely applicable, though further implementation and evaluation would be needed to verify this. Another limitation of this study was low survey participation, potentially predisposing to non-response bias. In future work, this could be addressed by offering Veterans the survey at the time of elastography. Surveys could also further explore potential patient barriers to screening and be administered with both completing patients and those who decline elastography at point of outreach. The COVID-19 pandemic presented a unique challenge to program development. Government-mandated lockdowns and travel restrictions made it difficult to schedule patients for in-person testing. High staff turnover coupled with the dedicated time commitment required for chart reviews was insurmountable for several sites during the pandemic, leading to delays and eventual inability to initiate the project at six of our sites. Providers noted that their time and resources were top barriers to study completion. Finally, with any screening program there is the potential for unintended negative consequences, including potential psychological and resource implications, that must be considered. While resources are an important consideration in any public health and screening program, we opted to use a free first pass test (FIB-4 labs are available using labs collected for other purposes) with conservative thresholds to identify patients for potential engagement. We also found that the patients reported benefiting from the education about liver health that was provided with elastography. Elastography is relatively inexpensive and comparable to other tests that are widely used for screening (e.g., lung cancer screening CT, colonoscopy), particularly given that the VHA system has VCTE machines available in most hospitals and the test itself takes about 15 min.

Due to the burden of chart reviews, we cannot currently recommend the described approach for all patients with risk factors for advanced liver disease. However, in response to the success of this program, we identified a VHA dashboard that could be sorted to identify patients more easily. Currently we are working to identify more efficient approaches to risk stratification using this dashboard. Future patient risk categorization may leverage natural language processing to identify Veterans most likely to benefit from elastography and should compare the effectiveness and cost of applying different thresholds and types of screening and confirmatory tests and assess impacts of such testing on long-term patient outcomes.

## Conclusion

Using a stepwise approach of FIB-4 score followed by elastography in Veterans with risk factors for cirrhosis was feasible and could be tailored according to site resources and preferences. This process was acceptable to patients and providers. Nearly half of the patients who completed elastography through this process met criteria for hepatology referral. A greater proportion of patients accepted offers for elastography when contacted by primary care providers who they knew, rather than by a hepatology clinic, highlighting the important role of primary care in cirrhosis screening efforts. This pilot allowed us to identify the tools and approaches that could address barriers to scaling cirrhosis screening for high-risk patients in the US.

## Supplementary Information

Additional file 1. Patient survey.

Additional file 2. Provider survey.

Additional file 3. Stepwise Approach to Population Cirrhosis Screening.

## Data Availability

The (quality improvement) datasets used and/or analyzed during the current study are available from the corresponding author on reasonable request.

## Abbreviations

AICD: Automated implantable cardioverter defibrillator
ALT: Alanine transaminase
AST: Aspartate aminotransferase
AUD: Alcohol Use Disorder
CAP: Controlled attenuation parameter
CCI: Charlson Comorbidity Index
CPT: Current Procedural Terminology
DM: Diabetes mellitus
FIB-4: Fibrosis-4
HCV: Hepatitis C infection
ICD: International Statistical Classification of Diseases and Related Health Probelems
IQR/M: Interquartile range/Median
kPa: Kilopascals
MASLD: Metabolic-associated steatotic liver disease
MRE: MRI elastography
NGHP: National Gastroenterology and Hepatology Program
PC: Primary care
PCP: Primary care physician
QI: Quality improvement
VCTE: Vibration controlled transient elastography
VHA: Veterans Health Administration
