# Supplementary material for: Screening high-risk Veterans for cirrhosis: taking a stepwise population health approach
Source: BMC Health Serv Res. 2025 Jan 29;25:168. doi: 10.1186/s12913-025-12216-8 (PMC11776120; doi:10.1186/s12913-025-12216-8)
Supplement: Supplementary file 1 — Additional file 1. Patient survey. [file 12913_2025_12216_MOESM1_ESM.docx]

**Supplementary File 1: Patient survey**

**Liver Health Screening Questionnaire**

| 1. **Overall, how satisfied were you with the liver health screening?**    - Very Dissatisfied    - Dissatisfied    - Neutral – not dissatisfied or satisfied    - Satisfied    - Very Satisfied | | | | | | |
| --- | --- | --- | --- | --- | --- | --- |
| **How much do you agree with these statements?** | | | | | | |
|  | **Strongly Disagree** | **Disagree** | **Neutral** | **Agree** | **Strongly Agree** | **Not Applicable** |
| 1. **My provider clearly explained the purpose of the screening procedure to me.** |  |  |  |  |  |  |
| 1. **The screening appointment was easy for me to schedule.** |  |  |  |  |  |  |
| 1. **The screening appointment was easy for me to attend.** |  |  |  |  |  |  |
| 1. **I felt comfortable during the screening procedure.** |  |  |  |  |  |  |
| 1. **The results of the procedure were easy to understand.** |  |  |  |  |  |  |
| 1. **I would recommend this screening procedure to other Veterans.** |  |  |  |  |  |  |

| 1. **Has this screening procedure changed how you think about your liver health?**  - Yes - No |
| --- |

**Please turn this page over to complete questions 9-11.**

| 1. **Will you take new actions to improve your liver health following this screening?**  - Yes - No | |
| --- | --- |
| **If yes, please briefly describe what actions you will take:** | |
|  |  |
|  | |
| 1. **How confident are you filling out forms by yourself?**  - Not at all - A little bit - Somewhat - Quite a bit - Extremely | |
| 1. **Please provide any additional comments or suggestions about the liver health screening:** | |
|  |  |
|  | |

**Thank you for your time and participation in VA’s Liver Health Screening project.**

**Please return this survey in the enclosed self-addressed envelope.**
