# Supplementary material for: Screening high-risk Veterans for cirrhosis: taking a stepwise population health approach
Source: BMC Health Serv Res. 2025 Jan 29;25:168. doi: 10.1186/s12913-025-12216-8 (PMC11776120; doi:10.1186/s12913-025-12216-8)
Supplement: Supplementary file 2 — Additional file 2. Provider survey. [file 12913_2025_12216_MOESM2_ESM.docx]

**Supplementary File 2: Provider survey**

**Liver Health Screening Questionnaire**

Thank you for taking the time to provide some brief feedback as a clinical partner providing care to Veterans at risk for cirrhosis.

| **VISN** (from dropdown) | **Facility** (from dropdown) |
| --- | --- |
| **Your Training:**  MD/PA/NP/RN/PharmD/DO  Other (please specify). | **Your Specialty: Please check all that apply**  Primary Care/Gastroenterology/Hepatology/Other (please specify). |
| **How many years have you been with the VA?**  (with year range options) | **Are you a designated site champion for the project?**  Yes No |

| 1. **Currently, how confident are you in your ability to:** | | | | | |
| --- | --- | --- | --- | --- | --- |
| - **Identify patients with undiagnosed cirrhosis** | 0 | 1 | 2 | 3 | 4 |
|  | Not at all confident | Slightly confident | Somewhat confident | Moderately confident | Very confident |
| - **Refer patients to FibroScan or elastography** | 0 | 1 | 2 | 3 | 4 |
|  | Not at all confident | Slightly confident | Somewhat confident | Moderately confident | Very confident |
| - **Interpret FibroScan or elastography results** | 0 | 1 | 2 | 3 | 4 |
|  | Not at all confident | Slightly confident | Somewhat confident | Moderately confident | Very confident |
| - **Talk to patients about FibroScan or elastography results** | 0 | 1 | 2 | 3 | 4 |
|  | Not at all confident | Slightly confident | Somewhat confident | Moderately confident | Very confident |

| 1. **The following are challenges or barriers to identifying patients with undiagnosed cirrhosis and linking them to hepatology care. Please check all that apply.** | |
| --- | --- |
| - - Provider/staff time   - Communication with patients   - Impact of COVID-19   - Communication between providers   - Other: __________________ | - - Patient co-pays   - Patient knowledge about liver disease   - Patient willingness to participate |
| 1. **Prior to this email, were you aware of a QI effort at your station to improve diagnosis of cirrhosis and link patients to hepatology (an Undiagnosed Cirrhosis initiative)?**   Yes (proceed to next questions)  No (skip to end) | |

| **Thank you for your participation in this QI initiative! We have a few additional questions about the project.**  Please select the response that best describes how much you agree or disagree with each statement below. For statements that don’t apply to your role, please select Not Applicable. | | | | | | | |
| --- | --- | --- | --- | --- | --- | --- | --- |
|  | **Strongly Agree** | **Agree** | **Neutral** | **Disagree** | **Strongly Disagree** |  | **Not Applicable** |
| 1. **I feel confident in my ability to assist with chart review to determine appropriate patients for screening.** |  |  |  |  |  |  |  |
| 1. **I feel confident in my ability to review project documents in CPRS.** |  |  |  |  |  |  |  |
| 1. **I feel confident in my ability to refer patients for testing.** |  |  |  |  |  |  |  |
| 1. **I feel confident in my ability to introduce the project to patients.** |  |  |  |  |  |  |  |
| 1. **I feel confident in my ability to communicate with the project champion.** |  |  |  |  |  |  |  |
| 1. **I would support wider implementation of undiagnosed cirrhosis screening in VHA.** |  |  |  |  |  |  |  |
| 1. **This screening process improved the quality of care for my patients.** |  |  |  |  |  |  |  |

| 1. **After participating with this project, I am better able to (check all that apply):**    - Identify Veterans with findings concerning for possible cirrhosis    - Communicate with specialty care    - Order tests to evaluate the stage of liver disease    - Discuss FibroScan or elastography with patients    - Determine which Veterans should be referred to specialty care for liver disease    - Other________________________________________    - None of the above |
| --- |

| 1. **What could we do to improve this process?** |
| --- |
| 1. **Please provide any other comments or feedback:** |

**Thank you for your time and for providing valuable feedback that helps inform liver care in VA!**
