# Supplementary material for: Screening high-risk Veterans for cirrhosis: taking a stepwise population health approach
Source: BMC Health Serv Res. 2025 Jan 29;25:168. doi: 10.1186/s12913-025-12216-8 (PMC11776120; doi:10.1186/s12913-025-12216-8)
Supplement: Supplementary file 3 — Additional file 3. Stepwise Approach to Population Cirrhosis Screening. [file 12913_2025_12216_MOESM3_ESM.docx]

**Supplementary File 3:** **Stepwise Approach to Population Cirrhosis Screening**

| Author | Year | Screening Inclusion | kPa cutoff | Follow-up Process |
| --- | --- | --- | --- | --- |
| Morling et al (33) | 2014 | Men and women aged 60–74 years  Type 2 DM, recruited by random selection from a register of patients with diabetes | ≥8.0 kPa | Five biomarkers (AST/ALT, APRI, ELF, FIB-4 and TE) were evaluated in the cohort at baseline, 1 year and 4 years; TE was done at 4 years only.  Any patients with abnormal results underwent a more comprehensive liver screen. |
| Harman et al (3) | 2015 | ALT ratio for alcohol risk groups  BARD score for other risk groups (DM or persistently raised ALT) | ≥8.0 kPa | TE at the primary care practice (if BMI >35 kg/m^2^, TE was done in the hospital using XL probe for better accuracy); All patients received nurse-led lifestyle intervention; Cases with elevated TE were then reviewed by a consulting hepatologist. |
| Harris et al (5) | 2019 | Hazardous alcohol use >14 units/week women and >21 units per week men  DM-by presence of a Read code  Obesity by BMI within past five years ≥30 kg/m^2^ for all patients non-Asian and ≥27 kg/m^2^ for patients of Asian ethnicity. | ≥8.0 kPa | TE scan in the primary care practice. Those with ≥8.0 kPa were then referred to see a hepatologist in the primary care practice for further evaluation. |
| Davyduke et al (32) | 2019 | Elevated ALT and/or steatosis on imaging  Age 16–65 years  Excluded heavy drinkers (women >4 drinks/day, men >7 drinks/day), but following RN screening did include patients with significant alcohol use. | ≥8.0 kPa | Patients meeting criteria were scheduled by PCP for a nurse led VCTE. Those with <8.0 kPa were counseled on lifestyle changes, discharged to PCP with recommendations and scheduled for reassessment in 2 years; Patients with >8.0 kPa or a failed VCTE were triaged to see the hepatologist.  Retrospective modeling to assess the impact of implementing a FIB-4 first strategy for triaging patients was then conducted using a threshold of 1.3 as low risk with no further liver assessment required. |
| Chalmers et al (4) | 2020 | AST/ALT ratio >0.8  Harmful alcohol use (>50 units/week for men and >35  units/week for women, or presence of Read codes related  to alcohol misuse).  Risk of NAFLD (a fatty liver index (FLI) ≥60, and (1) presence of obesity, type 2 diabetes, or metabolic syndrome, or (2) evidence of NAFLD on imaging). | ≥8.0 kPa | Patients meeting at least one screening criteria were scheduled for a TE clinic visit.  TE result is reported back to the GP with advice on interpretation and the following referral guidelines:   - <8 kPa should repeat TE in 5 years; - Between 8 kPa and 14.9 kPa were considered for hepatology referral or repeat TE in 3 years; - ≥15 kPa were referred to the local hepatology service. |
